# Lectures on Diseases and Injuries of the Ear

**Published:** 1873-12

**Authors:** 


### Books Reviewed.

*Lectures on Diseases and Injuries of the Ear, delivered at St. George's Hospital.* By W. B. Dalby, F. R. C. S ; M. B. Cantab, Aural Surgeon to the Hospital. Philadelphia: Lindsay & Blackiston, 1873; Buffalo: T. Butler & Son.

